# Untargeted metabolomics reveals distinct biomarkers and metabolic alterations in familial and non-genetic hypercholesterolemia in Saudi patients

**DOI:** 10.3389/fmed.2025.1670282

**Published:** 2025-09-26

**Authors:** Hadiah Bassam Al Mahdi, Noor Ahmad Shaik, Zuhier Awan, Hussam Daghistani, Faisal Alandejani, Kawthar Saad Alghamdi, Ahmad A. Obaid, Rawabi Zahed, Reem Nabil Hassan, Sherif Edris, Babajan Banaganapalli, Abdulrahman Mujalli

**Affiliations:** 1 Department of Biological Sciences, Faculty of Science, King Abdulaziz University, Jeddah, Saudi Arabia; 2 Department of Genetic Medicine, Faculty of Medicine, King Abdulaziz University, Jeddah, Saudi Arabia; 3 Princess Al-Jawhara Al-Brahim Centre of Excellence in Research of Hereditary Disorders, King Abdulaziz University, Jeddah, Saudi Arabia; 4 Department of Clinical Biochemistry, Faculty of Medicine, King Abdulaziz University, Jeddah, Saudi Arabia; 5 Regenerative Medicine Unit, King Fahd Medical Research Center, King Abdulaziz University, Jeddah, Saudi Arabia; 6 Department of Biology, College of Science, University of Hafr Al Batin, Hafar Al Batin, Saudi Arabia; 7 Department of Physiology, Faculty of Medicine, Umm Al-Qura University, Makkah, Saudi Arabia; 8 Department of Clinical Laboratory Sciences, Faculty of Applied Medical Sciences, Umm Al-Qura University, Makkah, Saudi Arabia

**Keywords:** familial hypercholesterolemia, untargeted metabolomics, KEGG database, biomarkers, cholic acid

## Abstract

**Background:**

Familial hypercholesterolemia (FH) and non-genetic hypercholesterolemia (HC) are both associated with elevated low-density cholesterol (LDL-C) levels, which increase the risk of cardiovascular disease. However, their underlying metabolic disturbances differ significantly. Untargeted metabolomics offers a powerful approach for identifying disease-specific metabolic signatures and potential biomarkers, thereby contributing to precision medicine applications.

**Methods:**

A high-resolution metabolomics analysis was performed using ultra-performance liquid chromatography coupled with quadrupole time-of-flight mass spectrometry (UPLC-Q-TOF/MS) on plasma samples from FH, HC, and healthy Saudi individuals. Differentially expressed metabolites were identified through univariate and multivariate analyses, followed by pathway enrichment analysis using the KEGG database.

**Results:**

Metabolic profiling revealed distinct alterations in bile acid biosynthesis and steroid metabolism pathways in FH. Cholic acid was significantly downregulated, while 17α-hydroxyprogesterone (17α-OHP) was significantly elevated in FH. In contrast, HC was characterized by increased uric acid and choline levels, along with dysregulation in oleic acid and linoleic acid metabolism. Notably, both FH and HC groups were dysregulated in Sphinganine, D-*α*-hydroxyglutaric acid, and pyridoxamine.

**Conclusion:**

This study demonstrates the utility of untargeted metabolomics in distinguishing FH from HC, identifying 17α-OHP and cholic acid as potential FH biomarkers, while uric acid and choline may serve as HC-specific metabolic markers. These findings provide new insights for personalized interventions, enhancing disease stratification and therapeutic decision-making between genetic and non-genetic hypercholesterolemia.

## 1 Introduction

Hypercholesterolemia is a metabolic disorder characterized by elevated low-density lipoprotein cholesterol (LDL-C), contributing significantly to atherosclerosis and cardiovascular diseases (CVDs) (1). Primary hypercholesterolemia includes familial hypercholesterolemia (FH, OMIM #143890), a monogenic disorder caused by variants in genes such as *LDLR*, *APOB*, and *PCSK9* (autosomal dominant), or *LDLRAP1* (autosomal recessive) (2, 3). These genetic alterations impair LDL-C clearance, leading to persistently high cholesterol levels from an early age and an increased risk of premature CVD. The global prevalence of FH is approximately 1 in 250, but it is substantially higher in the Gulf region, around 1 in 112, due to high consanguinity rates (4–7). Despite this, omics-based studies, particularly metabolomics, remain limited in Middle Eastern populations. In parallel, secondary or non-genetic hypercholesterolemia, which is driven by factors such as diet, obesity, and metabolic syndrome, may exhibit clinical features similar to those of inherited forms, making it difficult to distinguish between them using standard lipid profiling (1, 8, 9). Given that genetic variants can directly influence metabolic pathways, individuals with FH are likely to exhibit distinct metabolic signatures compared to those with secondary hypercholesterolemia (10). Understanding these differences is critical for accurate diagnosis, personalized risk assessment, and targeted treatment.

Untargeted metabolomics enables comprehensive profiling of small molecules, offering a powerful approach to identify disease-specific metabolic phenotypes and biomarkers that reflect both genetic and environmental influences on lipid metabolism (11, 12). Among various platforms, untargeted metabolomics using ultra-performance liquid chromatography coupled with quadrupole time-of-flight mass spectrometry (UPLC-Q-TOF/MS) offers high sensitivity and broad metabolite coverage, enabling detailed profiling of both the metabolome and lipidome (13, 14). This approach surpasses traditional lipid panels by uncovering disturbances in key metabolic pathways, such as in bile acid biosynthesis, steroid hormone metabolism, and oxidative stress, while simultaneously providing mechanistic insights into cholesterol-related pathologies like atherosclerosis, thereby facilitating biomarker discovery, improving disease classification, and advancing precision medicine strategies (15–17). A recent cross-sectional study conducted, utilizing LC–MS-based untargeted metabolomics, successfully identified unique metabolites associated with different FH forms. The analysis identified seven key discriminatory metabolites, including lithocholic acid (LCA), triacylglycerol TAG 52:2, 3-phenylpropionate, pipecolic acid, 3-indolepropionic acid, isocitric acid, and glycerophosphocholine (GPC) 38:5 (18).

Despite these advances, no studies to date have applied untargeted metabolomics to differentiate genetic and non-genetic forms of hypercholesterolemia in the Saudi population, which is characterized by unique genetic backgrounds and high rates of early-onset CVD. Therefore, this study aims to apply untargeted high-resolution metabolomics to distinguish between familial and secondary hypercholesterolemia in Saudi patients, identify disease-specific metabolic signatures, and uncover clinically relevant biomarkers. These insights are expected to advance disease classification and support the implementation of precision medicine strategies in hypercholesterolemia management.

Studying hypercholesterolemia in the Saudi population with its unique genetic background and high incidence of early-onset CVD presents a valuable opportunity to fill a key knowledge gap and advance precision medicine strategies tailored to the region. This study aims to apply untargeted high-resolution metabolomics to differentiate familial and non-genetic hypercholesterolemia in Saudi patients, identify specific metabolic signatures, and discover potential biomarkers for clinical use. These insights are expected to support precision medicine by improving disease classification and individualized treatment strategies.

## 2 Materials and methods

### 2.1 Study population

This study was approved by the Biomedical Ethics and Research Committee at King Abdulaziz University, Jeddah, Saudi Arabia (Reference Number: 220–22). Families were recruited from the Genetic Dyslipidemia and Familial Hypercholesterolemia Clinic at King Abdulaziz University Hospital, Jeddah, Saudi Arabia. The study included three groups of participants: familial hypercholesterolemia (FH), hypercholesterolemia (HC), and healthy controls (Healthy), consisting of both index patients and unaffected family members as controls.

### 2.2 Sample collection

A 3 mL blood EDTA tube was collected after 10–12 h of fasting, and only individuals without prior lipid-lowering treatment or medication were included. The groups were classified based on LDL-C levels determined through biochemical laboratory testing and genetic testing results (1, 8, 9). Participants were classified based on the results of biochemical and genetic testing, as reported previously (19).

The Healthy group included individuals with LDL-C levels below 100 mg/dL and no detected pathogenic variants in the FH-related genes. The HC consisted of individuals with LDL-C levels ranging from 130 to 159 mg/dL, without confirmed variants in the FH-related genes. The FH group included individuals with LDL-C levels ≥190 mg/dL and a confirmed pathogenic variant in the FH-related genes.

### 2.3 Metabolites extraction

For a plasma separation, blood was centrifuged at 2,000 × g for 15 min at 4 °C, and the supernatant was aliquoted (100–200 μL) into a sterile Eppendorf (EP) tube. Plasma samples were then stored at −80 °C until further sample processing.

For metabolite extraction, 100 μL of plasma was mixed with 700 μL of extraction solvent containing an internal standard (Methanol: Acetonitrile: Water, 4:2:1, v/v/v). The mixture was vortexed for 1 min and incubated at −20 °C for 2 h, then centrifuged at 25,000 × g at 4 °C for 15 min. After centrifugation, 600 μL of the supernatant was transferred to a new EP tube and dried using a vacuum concentrator. The dried extracts were reconstituted in 180 μL of Methanol: Water (1:1, v/v), vortexed for 10 min, and centrifuged again at 25,000 × g at 4 °C for 15 min to remove any insoluble debris. The final supernatants were transferred to fresh EP tubes for downstream metabolomic analysis.

### 2.4 Liquid chromatography-mass spectrometry (LC–MS) workflow

The metabolomics analysis was conducted in collaboration with the Beijing Genomics Institute (BGI). Metabolite separation and detection were performed using a Waters UPLC I-Class Plus system (Waters, USA) coupled with a Q Exactive Orbitrap high-resolution tandem mass spectrometer (Thermo Fisher Scientific, USA). Chromatographic separation was carried out using a Waters ACQUITY UPLC BEH C18 column (1.7 μm, 2.1 mm × 100 mm, Waters, USA), with the column temperature maintained at 45 °C.

The mobile phase was prepared based on the ionization mode. In positive ion mode, the mobile phase consisted of 0.1% formic acid (A) and acetonitrile (B), whereas in negative ion mode, the mobile phase contained 10 mM ammonium formate (A) and acetonitrile (B). A gradient elution program was applied with an initial 0 to 1 min 2% B, linearly increasing to 98% B from 1 to 9 min, maintaining 98% B from 9 to 12 min, then reverting to 2% B at 12.1 min, followed by equilibration at 2% B for 12.1 to 15 min. The flow rate was maintained at 0.35 mL/min, and the injection volume was 5 μL.

Mass spectrometric analysis was performed using full scan and tandem MS (MS/MS). The scan range was set from 70 to 1,050 m/z, with a resolution of 70,000 for full MS scans. The Automatic Gain Control (AGC) target was configured to 3 × 106, with a maximum ion injection time of 100 ms. For MS/MS fragmentation, the top 3 precursor ions were selected per cycle with an injection time of 50 ms, a resolution of 17,500, and the AGC was 1 × 105. The stepped normalized collision energy was 20, 40, and 60 eV to enhance fragmentation efficiency. Electrospray ionization (ESI) settings were optimized as follows: the sheath gas flow rate was set to 40, and the auxiliary gas flow rate was set to 10. The spray voltage was adjusted to 3.80 kV for positive ion mode and 3.20 kV for negative ion mode. The capillary temperature was maintained at 320 °C, while the auxiliary gas heater temperature was set to 350 °C.

### 2.5 Peak area and metabolites identification

The generated mass spectrometry data were imported and processed using Compound Discoverer v3.3 (Thermo Fisher Scientific, USA)^1^. To ensure comprehensive metabolite identification, multiple databases, including BGI Metabolome Database (BMDB), mzCloud, and ChemSpider (HMDB, KEGG, LipidMaps) databases, were used for metabolite peak areas extraction and metabolite identification.

### 2.6 Data processing

The results file generated from Compound Discoverer v3.3 was imported into MetaX software (20) for data preprocessing and statistical analysis. To minimize technical variability and enhance the reliability of metabolomic data, the following preprocessing steps were implemented. Probabilistic Quotient Normalization (PQN) was applied to normalize metabolite intensities by generating a reference vector based on ion intensity distribution across all samples and adjusting each sample accordingly (21). Additionally, Quality Control-based Robust LOESS Signal Correction (QC-RLSC) was implemented to correct batch effects using local polynomial regression fitting based on QC samples to enhance signal consistency (22). To ensure high-quality and reproducible data for downstream analysis, metabolites with a Coefficient of Variation (CV) > 30% across QC samples were considered unstable and excluded.

The identified substances were classified into different credibility levels based on available matching criteria, including MS1 molecular weight, MS2 fragment spectra, column retention time, and the presence of reference standards. Level 1 represents the highest credibility, where substances are accurately identified using both the standard databases and laboratory data. Level 2 includes substances with a structural formula that matches the standard databases. Level 3 substances partially match the database but require further validation. Level 4 substances are identified solely based on accurate MS1 molecular weight matching the database. Level 5 represents the lowest credibility, with no matches or identification results available in the database. The credibility of identification decreases progressively from Level 1 to Level 5.

### 2.7 Overall metabolites analysis

To gain more insight into the biological functions and classifications of the identified metabolites from levels 1 to 4. The identified metabolites were analyzed and annotated using the Kyoto Encyclopedia of Genes and Genomes (KEGG) and the Human Metabolome Database (HMDB). The analysis of identified metabolites was categorized according to their Super Pathway. However, for lipid molecules, the Sub Pathway classification was used instead.

### 2.8 Differential metabolite screening

Univariate analysis was conducted to assess the statistical significance of differences in metabolite expression between comparison groups, with fold change (FC) calculated as the ratio of metabolite levels and t-tests used for statistical evaluation. Metabolites were considered differentially expressed if they met the threshold of FC ≥ 1.2 or ≤ 0.83 and adjusted *p*-value < 0.05. The log₂FC transformation was applied to normalize data distribution and enhance interpretability. Statistical significance was determined using *t*-tests, and adjusted *p*-values were adjusted for multiple comparisons using the Benjamini-Hochberg method to control the False Discovery Rate (FDR). The log₂FC and adjusted *p*-value were used for downstream statistical analysis. The volcano plots were generated to visualize differentially expressed metabolites (DEMs), highlighting significantly upregulated and downregulated metabolites based on their fold changes and adjusted *p*-values. Metabolites with a high absolute log₂ fold change and adjusted p-value were considered statistically significant. Heatmaps were generated to illustrate clustering patterns of DEMs between experimental groups.

In multivariate analysis, the orthogonal partial least squares discriminant analysis (OPLS-DA) is used to develop predictive models and identify key metabolites by distinguishing between systematic and orthogonal variance. This enhances classification accuracy and provides a robust evaluation of metabolic distinctions.

### 2.9 Correlation analysis

Chord diagrams were constructed to evaluate metabolite co-regulation relationships based on the Spearman correlation coefficient (|*r*| > 0.8, *p* < 0.05). These diagrams provide an intuitive visualization of metabolite-metabolite interactions, distinguishing between synergistic (positive correlations) and antagonistic (negative correlations), with color variations representing different correlation patterns. The primary objective of differential metabolite correlation analysis was to assess the consistency of metabolite fluctuations and determine their interdependence.

### 2.10 Pathway enrichment analysis

Metabolic pathway enrichment analysis of DEMs was performed using the KEGG database to identify significantly altered pathways and interpret biological phenotypes. Pathways with adjusted *p*-value < 0.05 were considered significantly enriched, highlighting key metabolic shifts. To provide a comprehensive view of metabolic alterations, the Differential Abundance (DA) Score method was applied to evaluate cumulative metabolite changes within specific pathways. The DA score ranges from −1 to +1, where +1 indicates complete upregulation of a pathway, 0 represents no significant change, and −1 denotes complete downregulation. Intermediate values reflect partial upregulation or downregulation based on the proportion of upregulated and downregulated metabolites within the pathway.

## 3 Results

### 3.1 Sample description

In this study, 16 fasting plasma samples were analyzed and categorized into three groups: FH (*n* = 7), HC (*n* = 4), and Healthy (*n* = 5). The average LDL-C levels were 367.3 ± 101.8 mg/dL in the FH group. This level was approximately 3.9 times higher than that of the Healthy group (94.6 ± 11.4 mg/dL) and 2.6 times higher than that of the HC group (139.2 ± 4.5 mg/dL). Genetically, all participants in the FH group carried a heterozygous mutation in the *LDLR* gene (c.2416dupG and c.103C > T), while other groups were negative for FH-related genes. For comparative analysis, three pairwise comparisons were conducted: FH vs. Healthy, HC vs. Healthy, and FH vs. HC to assess metabolic variations across groups. Sample characteristics, including LDL-C measurement, are detailed in the Supplementary Table 1.

### 3.2 Chromatographic and mass spectrometric analysis

Untargeted metabolomics profiling in both positive and negative ion modes revealed a diverse range of metabolites across the 15-min chromatographic run. As shown in the Base Peak Chromatograms (BPC) (Supplementary Figure 1), the wide distribution of peaks in both ionization modes confirms broad metabolite coverage and efficient chromatographic separation.

In positive ion mode, prominent features were observed at 7.36, 9.71, and 12.75 min, corresponding to abundant hydrophobic metabolites, while earlier peaks (at 0.88 and 2.67 min) indicated the presence of polar compounds. The negative mode captured a distinct profile of acidic and polar metabolites, with major peaks detected at 9.73, 10.32, and 12.83 min.

### 3.3 Metabolites detection and identification

**Figure 1:**
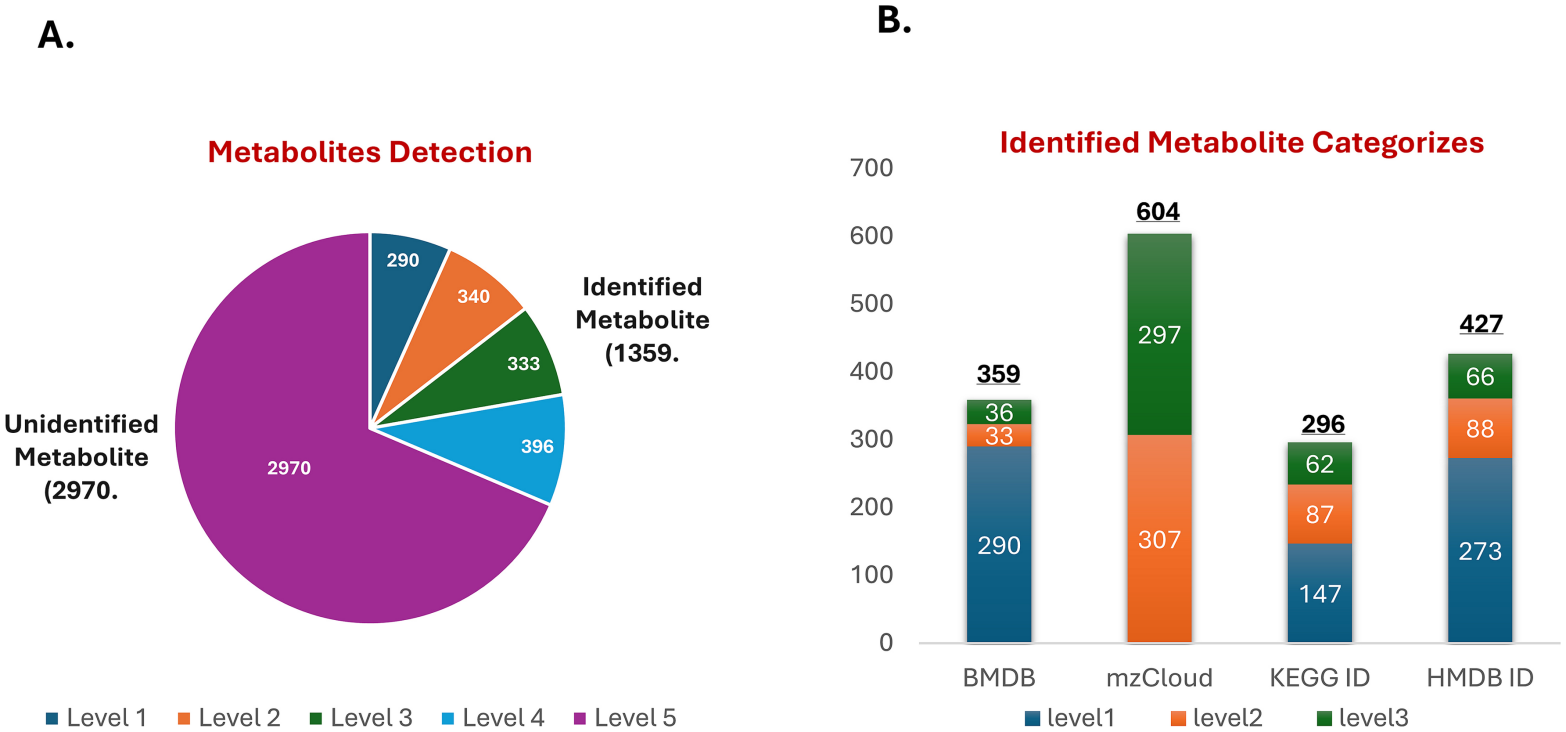
Metabolite identification. **(A)** The pie chart illustrates the proportion of identified metabolites (1,359) versus unidentified metabolites (2,970). Identified metabolites are classified into five levels based on credibility, with Level 1 representing the highest credibility and Level 5 the lowest. **(B)** The bar chart displays the distribution of identified metabolites across different databases (BMDB, mzCloud, KEGG, and HMDB), with each bar segmented by credibility levels (Level 1 to Level 3), demonstrating the contribution of each database to metabolite classification and functional annotation.

After data preprocessing, a total of 4,850 metabolites were detected, of which 4,329 metabolites had a CV ≤ 30%. These metabolites were mapped and classified based on their level of credibility as described in the methods (section 2.6). Figure 1 shows that only 1,359 substances were mapped from Level 1 to Level 4, considered identified, while the remaining metabolites were classified as Level 5 (unidentified). Furthermore, identified metabolites with putative names were annotated using the HMDB and KEGG databases and subsequently classified into four major categories: lipids, phytochemical compounds, biologically active compounds, and others. Among these, lipids (*n* = 132) and amino acids, peptides, and analogues (n = 64) represented the two largest classes. Detailed metabolite classifications and KEGG pathway annotations are provided in Supplementary Figure 2.

### 3.4 Differential metabolites screening and analysis

**Figure 2:**
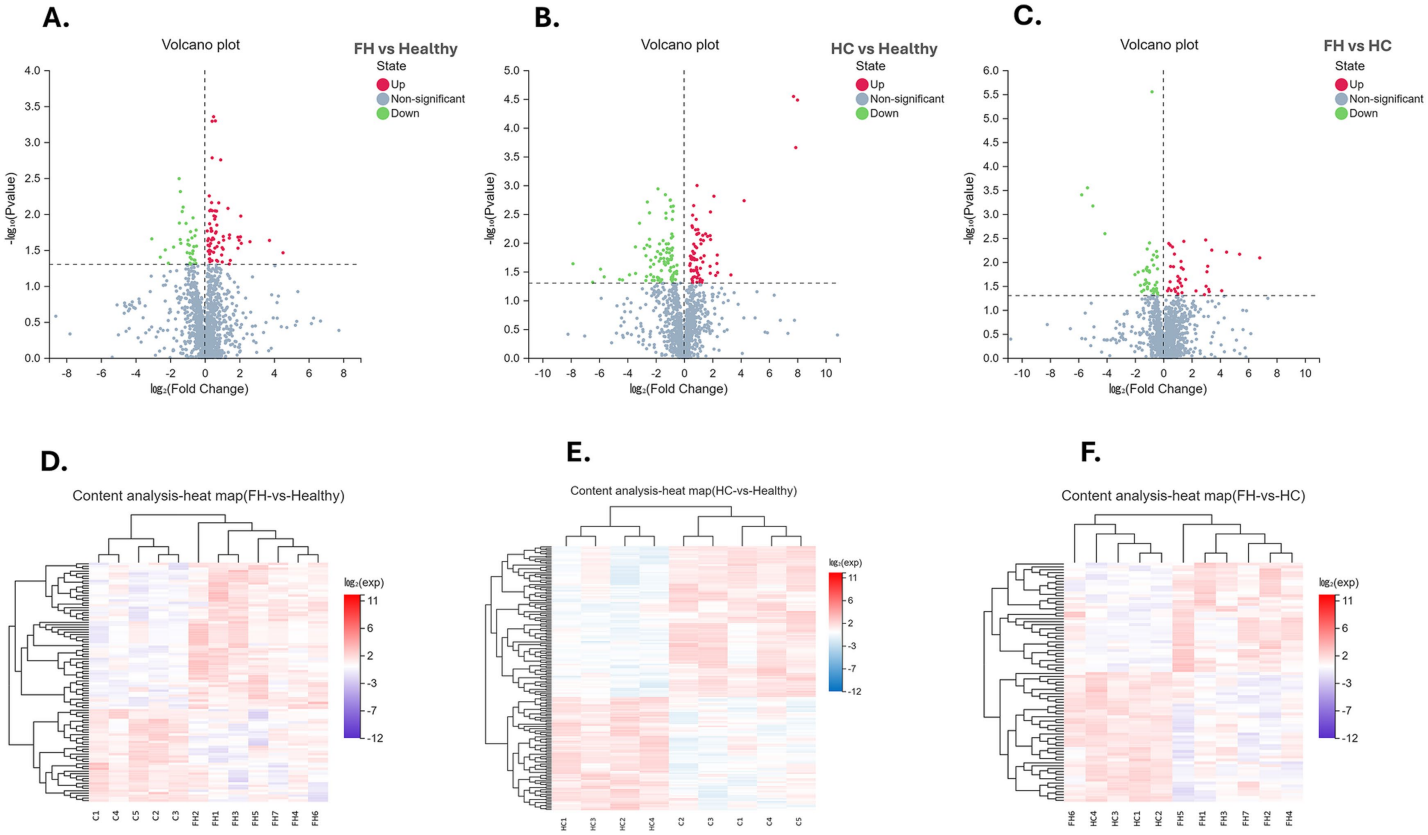
Visualization of differential metabolite analysis across comparison groups. **(A–C)** Volcano plots for FH vs. Healthy (left), HC vs. Healthy (middle), and FH vs. HC (right), illustrating the relationship between log₂ (Fold Change) (X-axis) and −log₁₀ (*p*-value) (Y-axis). Metabolites are classified as upregulated (red), downregulated (green), or non-significant (blue). **(D–F)** Heatmaps displaying the expression patterns of differentially expressed metabolites across FH vs. Healthy (left), HC vs. Healthy (middle), and FH vs. HC (right). Hierarchical clustering reveals metabolic variations between groups. The color scale represents log₂-transformed expression values, where red indicates upregulation and blue indicates downregulation.

**Figure 3:**
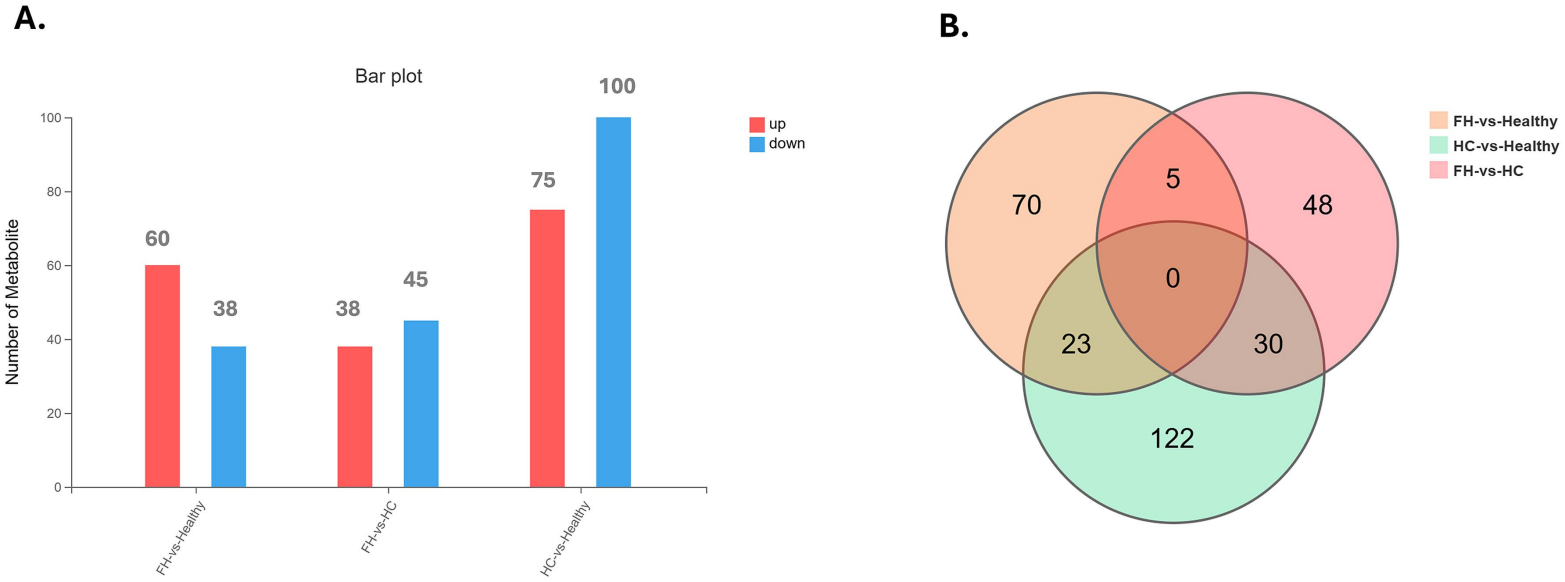
Distribution of differential metabolites across comparison groups. **(A)** Bar plot displaying the number of upregulated (red) and downregulated (blue) metabolites in FH vs. Healthy, FH vs. HC, and HC vs. Healthy groups. **(B)** Venn diagram illustrating the overlap of differentially expressed metabolites among the three comparison groups, highlighting both shared and unique metabolites.

Univariate analysis across the three groups identified DMEs and revealed distinct distribution patterns (Figure 2). In the FH vs. Healthy group, 98 DEMs were identified, with 60 upregulated and 38 downregulated. For the HC vs. Healthy group, 175 DEMs were identified, comprising 75 upregulated and 100 downregulated. Finally, in the FH vs. HC group, 83 DEMs were identified, including 38 upregulated and 45 downregulated (Figure 3). The significant DEMs annotated with HMDB and KEGG IDs were included in the enrichment analysis (Supplementary Table 2).

We then performed a multivariate statistical analysis using OPLS-DA, which revealed clear ellipses among the FH, HC, and Healthy groups.

**Figure 4:**
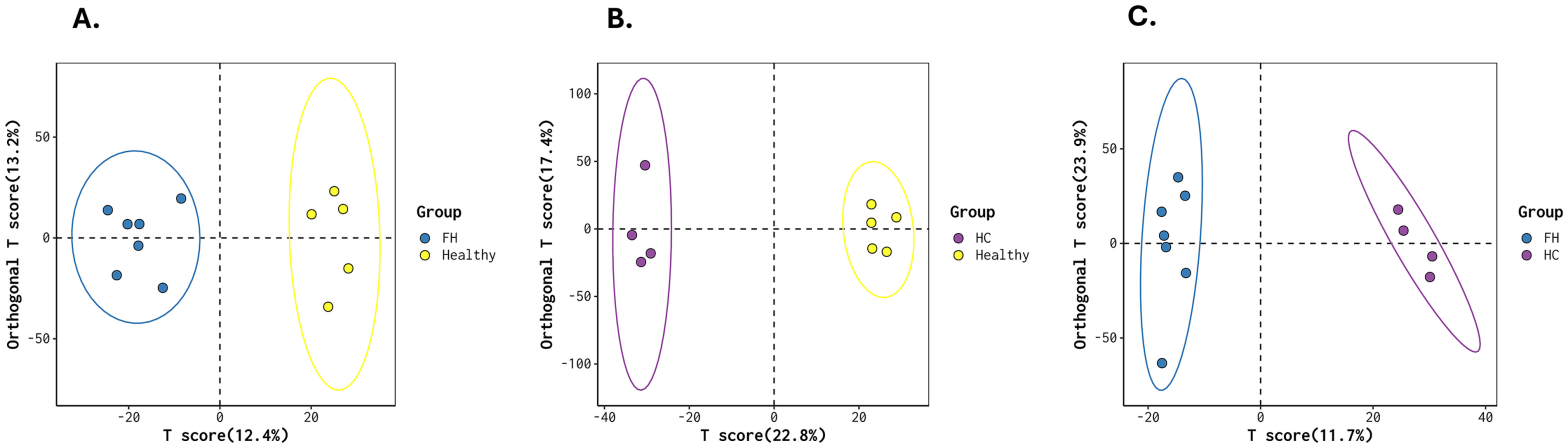
OPLS-DA score plots for metabolic differentiation across groups. **(A)** FH vs. Healthy, **(B)** HC vs. Healthy, and **(C)** FH vs. HC, illustrating distinct clustering patterns that highlight metabolic differences between groups. The separation of ellipses indicates variations in metabolic profiles across the groups.

The FH vs. Healthy comparison showed a T score of 12.4% and an Orthogonal T score of 13.2%. The HC vs. Healthy comparison had a T score of 22.8% and an Orthogonal T score of 17.4%. The FH vs. HC comparison showed a T score of 11.7% and an Orthogonal T score of 12.9% (Figure 4).

### 3.5 DEMs co-regulation relationships

**Figure 5:**
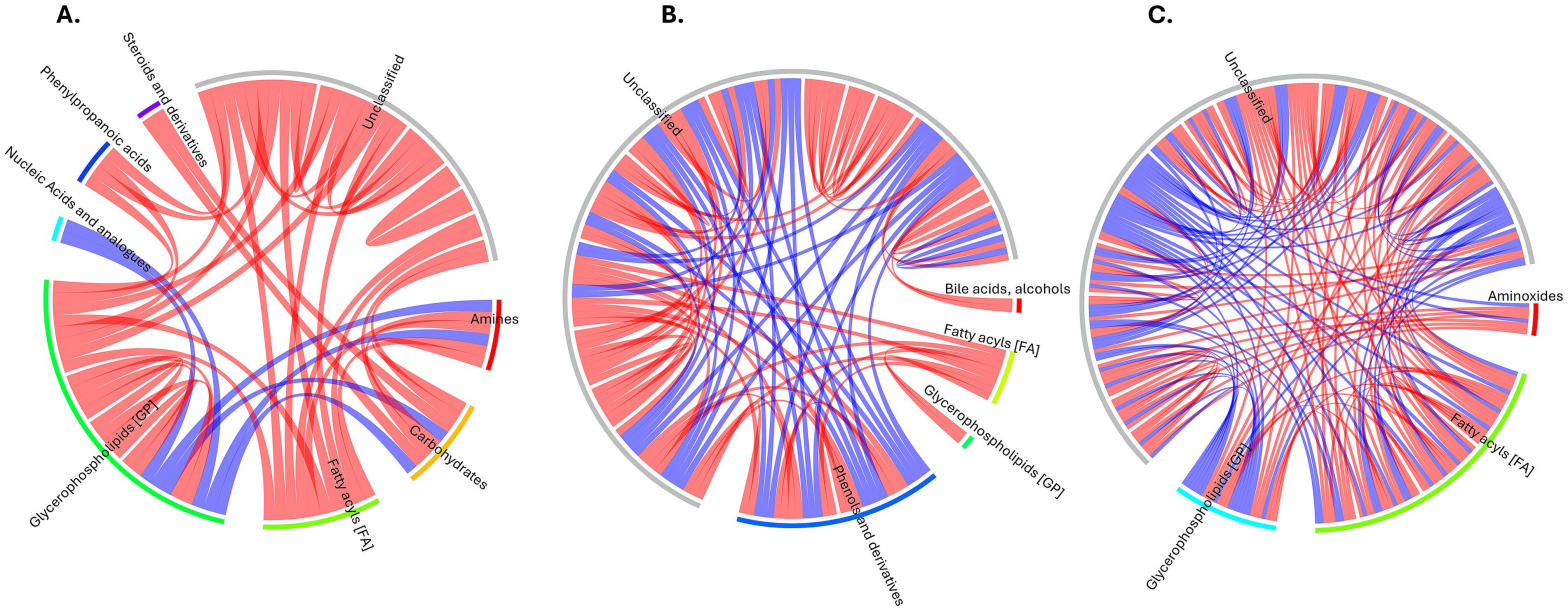
Correlation chord diagrams of metabolite relationships for comparison groups. **(A)** FH vs. Healthy. **(B)** FH vs. HC. **(C)** HC vs. Healthy. Color Key: Red: Positive correlations. Blue: Negative correlations. Connections represent significant correlations (*p* < 0.05) between metabolite pairs.

The correlation analysis revealed distinct patterns of metabolite interactions across the three comparison groups (Figure 5). In the FH vs. Healthy comparison (Figure 5A), strong positive intra-class correlations were observed among glycerophospholipids and steroid derivatives, suggesting a tightly regulated lipid metabolic network in individuals with FH. In contrast, negative correlations were noted between glycerophospholipids and amino sugar-related metabolites, indicating a possible dysregulation in membrane lipid signaling and transport. The FH vs. HC analysis demonstrated more divergent correlation patterns, where glycerophospholipids showed both positive and negative correlations with fatty acyls and bile acid derivatives (Figure 5B). These findings may reflect distinct bile acid biosynthesis, lipid remodeling, and oxidative stress response pathways that characterize genetically driven FH compared to HC. In the HC vs. Healthy group, a broader range of negative correlations was found across fatty acyls and glycerophospholipids, consistent with disrupted lipid regulation and systemic metabolic imbalance in HC (Figure 5C). These findings highlight the distinct metabolic network reorganization observed between FH and HC, emphasizing the role of lipid subclass-specific interactions as key contributors to the pathophysiology of different forms of hypercholesterolemia.

### 3.6 Pathway enrichment analysis

**Figure 6:**
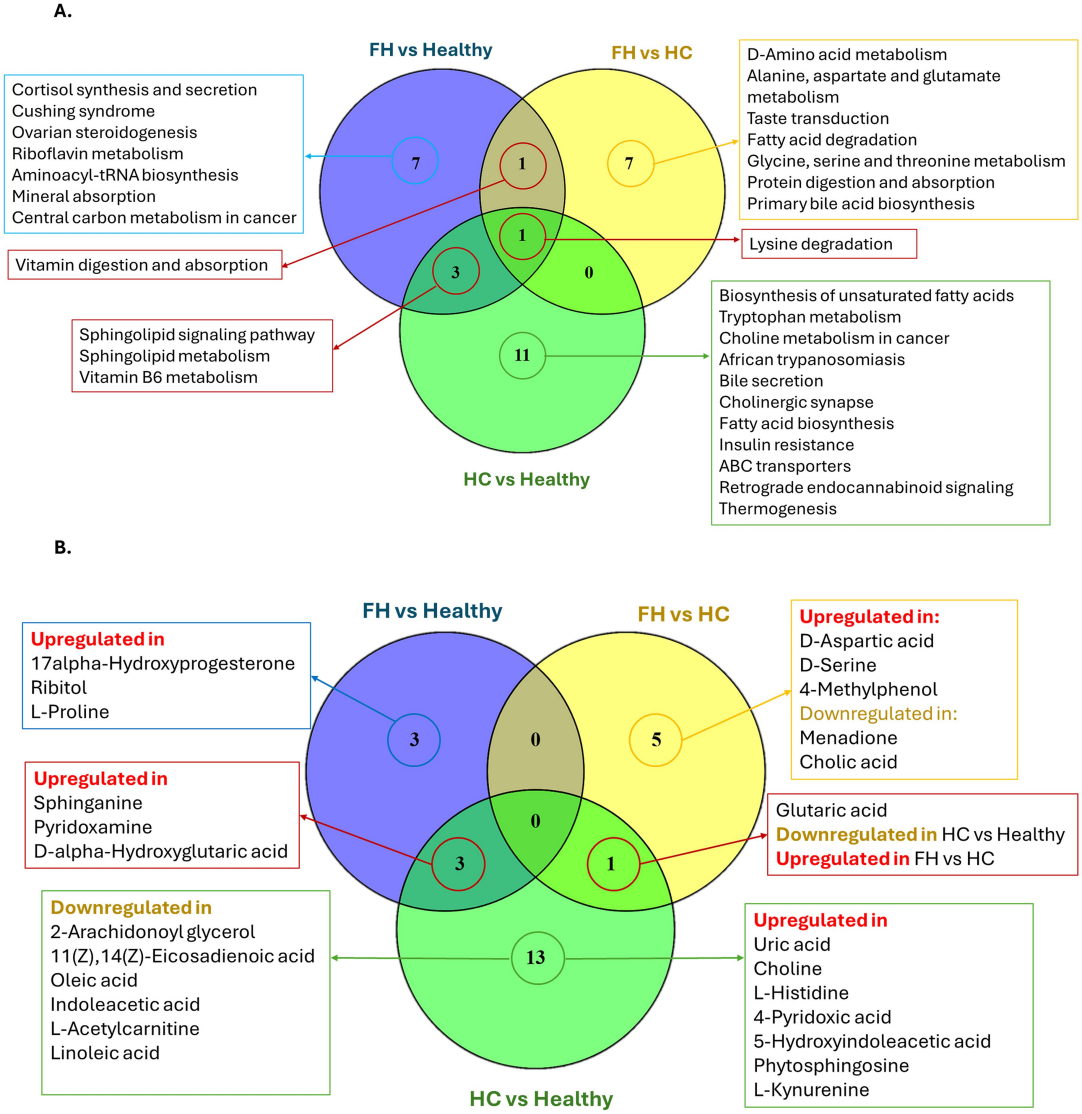
Shared and unique pathways and metabolites among comparison groups. **(A)** Venn diagram showing the number of significant metabolic pathways (adjusted p-value < 0.05) shared and unique among the three pairwise comparisons: FH vs. Healthy, FH vs. HC, and HC vs. Healthy. **(B)** Venn diagram displaying the significant metabolites contributing to those pathways, categorized by their regulation status (upregulated or downregulated) in each comparison.

Metabolic pathway enrichment analysis was performed using the KEGG database to identify significantly altered pathways across the comparison groups. The top enriched pathways were visualized using a bubble plot in Figure 6, with detailed results presented in Table 1 and Supplementary Table 3.

**Table 1:** KEGG pathway enrichment analysis across comparison groups.

Pathway ID	Pathway	Hit	Count	Adjusted *p*-value	KEGG Names	KEGG IDs
FH vs. Healthy						
map04071	Sphingolipid signaling pathway	1	10	0.0113	Sphinganine	C00836
map00600	Sphingolipid metabolism	1	11	0.0124	Sphinganine	C00836
map04927	Cortisol synthesis and secretion	1	12	0.0136	17alpha-Hydroxyprogesterone	C01176
map04934	Cushing syndrome	1	13	0.0147	17alpha-Hydroxyprogesterone	C01176
map04913	Ovarian steroidogenesis	1	23	0.0258	17alpha-Hydroxyprogesterone	C01176
map00740	Riboflavin metabolism	1	24	0.0269	Ribitol	C00474
map00970	Aminoacyl-tRNA biosynthesis	1	24	0.0269	L-Proline	C00148
map04978	Mineral absorption	1	29	0.0325	L-Proline	C00148
map00750	Vitamin B6 metabolism	1	29	0.0325	Pyridoxamine	C00534
map04977	Vitamin digestion and absorption	1	30	0.0336	Pyridoxamine	C00534
map05230	Central carbon metabolism in cancer	1	37	0.0412	L-Proline	C00148
map00310	Lysine degradation	1	45	0.0499	D-alpha-Hydroxyglutaric acid	C01087
HC vs. Healthy						
map00600	Sphingolipid metabolism	2	11	0.0004	Sphinganine; Phytosphingosine	C00836 C12144
map01040	Biosynthesis of unsaturated fatty acids	3	69	0.0008	Oleic acid; Linoleic acid; 11(Z),14(Z)-Eicosadienoic acid	C00712 C01595 C16525
map00380	Tryptophan metabolism	3	83	0.0013	L-Kynurenine; Indoleacetic acid;5-Hydroxyindoleacetic acid	C00328 C00954 C05635
map00750	Vitamin B6 metabolism	2	29	0.0026	4-Pyridoxic acid; Pyridoxamine	C00847 C00534
map00310	Lysine degradation	2	45	0.0062	D-alpha-Hydroxyglutaric acid; Glutaric acid	C01087 C00489
map05231	Choline metabolism in cancer	1	5	0.0131	Choline	C00114
map05143	African trypanosomiasis	1	7	0.0183	L-Kynurenine	C00328
map04976	Bile secretion	2	93	0.0249	Uric acid; Choline	C00366 C00114
map04071	Sphingolipid signaling pathway	1	10	0.0260	Sphinganine	C00836
map04725	Cholinergic synapse	1	10	0.0260	Choline	C00114
map00061	Fatty acid biosynthesis	1	12	0.0311	Oleic acid	C00712
map04931	Insulin resistance	1	12	0.0311	L-Acetylcarnitine	C02571
map02010	ABC transporters	2	118	0.0385	L-Histidine; Choline	C00135 C00114
map04723	Retrograde endocannabinoid signaling	1	15	0.0388	2-Arachidonoyl glycerol	C13856
map04714	Thermogenesis	1	17	0.0438	2-Arachidonoyl glycerol	C13856
FH vs. HC						
map00470	D-Amino acid metabolism	2	65	0.0011	D-Aspartic acid; D-Serine	C00402 C00740
map00250	Alanine, aspartate and glutamate metabolism	1	28	0.0216	D-Aspartic acid	C00402
map04977	Vitamin digestion and absorption	1	30	0.0231	Menadione	C05377
map04742	Taste transduction	1	32	0.0246	D-Serine	C00740
map00071	Fatty acid degradation	1	39	0.0299	Glutaric acid	C00489
map00260	Glycine, serine and threonine metabolism	1	44	0.0337	D-Serine	C00740
map00310	Lysine degradation	1	45	0.0344	Glutaric acid	C00489
map04974	Protein digestion and absorption	1	46	0.0352	4-Methylphenol	C01468
map00120	Primary bile acid biosynthesis	1	47	0.0359	Cholic acid	C00695

Shared and unique pathways are illustrated in Figure 6A. The Venn diagram showed the shared metabolites between the comparison groups (Figure 6B). Interestingly, the lysine degradation pathway (KEGG: map00310) was consistently enriched across all three comparative groups. In the FH vs. Healthy comparison, the pathway was significantly altered (adjusted *p*-value = 0.0499) due to the upregulation of D-*α*-hydroxyglutaric acid. In the HC vs. Healthy group, this pathway exhibited stronger significance (adjusted *p*-value = 0.0062) and involved two metabolites: D-*α*-hydroxyglutaric acid and glutaric acid. However, glutaric acid displayed opposite expression in the two comparisons: it was downregulated in HC vs. Healthy, but upregulated in FH vs. HC, where the lysine degradation pathway remained significant (adjusted *p*-value = 0.0344). The sphingolipid signaling pathway (KEGG: map04071) and sphingolipid metabolism pathway (KEGG: map00600) were significantly enriched in both FH and HC groups compared to healthy controls. In the FH vs. Healthy comparison, these pathways were enriched with sphinganine (KEGG: C00836) (adjusted *p*-value = 0.0113 and 0.0124, respectively). In contrast, in the HC vs. Healthy comparison, enrichment was driven by elevated levels of sphinganine and phytosphingosine (adjusted *p*-value = 0.0260 and 0.0004, respectively).

**Figure 7:**
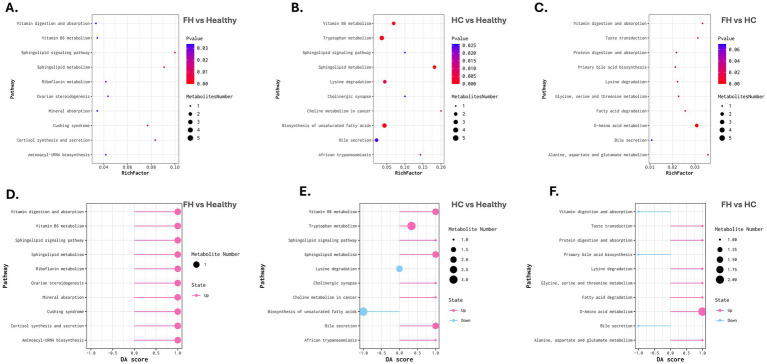
Metabolic pathway enrichment and differential abundance analysis across comparison groups. **(A–C)** Bubble plots representing metabolic pathway enrichment for FH vs. Healthy (left), HC vs. Healthy (middle), and FH vs. HC (right). The X-axis denotes the Rich Factor, while the Y-axis lists enriched pathways. Bubble size corresponds to the number of metabolites mapped to each pathway, and color intensity represents statistical significance (adjusted p-value), with darker colors indicating higher significance. **(D–F)** Differential Abundance (DA) Score plots for FH vs. Healthy (left), HC vs. Healthy (middle), and FH vs. HC (right). The X-axis represents the DA score, indicating the direction and magnitude of pathway regulation. Pink bars represent upregulated pathways, while blue bars indicate downregulated pathways. Bubble size corresponds to the number of metabolites associated with each pathway.

In the FH vs. Healthy comparison, the cortisol synthesis and secretion pathway (KEGG: map04927) was significantly enriched (adjusted *p*-value = 0.0136), with upregulation of D-*α*-hydroxyglutaric acid (Figures 7A,D). In the HC vs. Healthy comparison, significant alterations were observed in the bile secretion pathway (KEGG: map04976, adjusted *p*-value = 0.0249) and insulin resistance pathway (KEGG: map04931, adjusted *p*-value = 0.0311), accompanied by upregulation of uric acid, choline, and L-acetylcarnitine. Meanwhile, the fatty acid biosynthesis pathway (KEGG: map00061, adjusted *p*-value = 0.0311) was downregulated, reflected by decreased oleic and linoleic acid levels (Figures 7B,E).

The FH vs. HC analysis revealed significant enrichment in the primary bile acid biosynthesis pathway (KEGG: map00120, adjusted *p*-value = 0.0359), with downregulation of cholic acid (Figures 7C,F).

## 4 Discussion

Familial hypercholesterolemia is a metabolic disorder associated with a significantly increased risk of cardiovascular diseases and other health complications (23). Metabolomics, the comprehensive study of metabolites within biological systems, has emerged as a powerful tool in elucidating hyperlipidemic conditions characterized by elevated lipid levels in the bloodstream (24, 25). This approach not only enables the identification of novel biomarkers for early diagnosis but also provides insights into the efficacy of therapeutic interventions (17, 26). In this study, untargeted metabolomics revealed distinct metabolic alterations in both FH patients carrying LDLR variants (c.2416dupG, c.103C > T) and in individuals with HC, compared to healthy controls. KEGG pathway enrichment analysis (adjusted *p* < 0.05) showed significant disruptions in lipid metabolism across both groups. Although both FH and HC are characterized by elevated LDL-C levels, their underlying metabolic mechanisms differ. This distinction is clinically important, as FH is a monogenic disorder caused by inherited variants in lipid-regulating genes, whereas HC typically results from environmental factors, dietary habits, and broader metabolic dysregulation (1, 8, 9).

The FH group, when compared to healthy controls, exhibited significant enrichment in three metabolic pathways, including cortisol synthesis and secretion, Cushing syndrome, and ovarian steroidogenesis, primarily driven by elevated levels of 17α-hydroxyprogesterone (17α-OHP). As cholesterol is the precursor for all steroid hormones, these findings suggest FH caused by LDLR variants may prompt alternative tissues, such as the adrenal glands, cells can maintain high intracellular cholesterol by increasing cholesterol uptake via HDL pathways via scavenger receptors (27) and under sustained ACTH stimulation (28) which together upregulate steroidogenic enzymes such as CYP17A1 (17α-hydroxylase) (29, 30); consequently, the heightened steroidogenic flux can exceed the capacity of downstream steps like 21-hydroxylation by CYP21A2, leading to a bottleneck that causes accumulation of precursor steroids, notably 17α-OHP. Previous studies show that Elevated LDL enhances adrenal steroidogenesis by increased ACTH stimulation, commonly associated with metabolic stress in FH, resulting in elevated 17α-OHP levels due to a bottleneck at the CYP21A2 enzyme step (31–33). Clinically, 17α-OHP is a key diagnostic biomarker for congenital adrenal hyperplasia (34–36). In addition, elevated 17α-OHP can lead to an increased risk of cardiovascular (37). These alterations may contribute to broader metabolic and endocrine dysregulation in FH patients, particularly in the context of our study, where the majority of participants were female, suggesting potential sex-specific metabolic responses. The upregulation of 17α-OHP highlights a potential crosstalk between lipid metabolism and adrenal steroidogenesis in FH patients, and this metabolite is considered a secondary metabolite.

On the other hand, comparisons of FH and HC patients revealed a significant dysregulation in primary bile acid biosynthesis, showing that cholic acid levels were significantly downregulated in FH (38, 39). This indicates impaired primary bile acid biosynthesis, likely due to increased intracellular cholesterol that suppresses bile acid synthesis via negative feedback on CYP7A1 (40). Disruption of bile acid metabolism in FH may hinder lipid digestion and feedback regulation, contributing to further cholesterol accumulation (41–43). Moreover, the bile acid impairment observed in familial hypercholesterolemia highlights a significant therapeutic gap. Traditional bile acid sequestrants (BASs), such as cholestyramine, may be insufficient. Emerging biopolymer-based BASs could provide improved FH management by modulating both cholesterol and bile acid metabolic pathways (44–46). These findings indicate that cholic acid has the potential to serve as a discriminative biomarker between familial and non-genetic forms of hypercholesterolemia.

In the HC group, there was a significant downregulation in the biosynthesis pathways of unsaturated fatty acids (UFAs), including oleic acid, linoleic acid, and 11(Z),14(Z)-eicosadienoic acid, alongside alterations in insulin resistance-related metabolites such as L-acetylcarnitine. It is plausible that these shifts reflect lipid accumulation and impaired fatty acid oxidation, both of which are known to contribute to insulin resistance and metabolic inflexibility (47). These UFAs, particularly monounsaturated (MUFAs) and polyunsaturated fatty acids (PUFAs), are vital for cholesterol transport and CVD prevention (48–51). Reduced levels may be driven by insulin resistance, which suppresses stearoyl-CoA desaturase-1 (SCD1), the enzyme converting saturated fatty acids into MUFAs, including oleic acid (52, 53). Concurrently, insulin resistance and impaired efficient fatty acid oxidation, evidenced by accumulation of *β*-oxidation intermediates like L-acetylcarnitine (54). These findings suggest that dietary interventions and metabolic therapies targeting fatty acid metabolism and insulin resistance may be especially beneficial for HC patients, more so than for those with FH.

HC patients also showed upregulation in the Bile secretion pathway with elevated levels of uric acid and choline, both of which are involved in cholesterol metabolism and inflammatory responses. Elevated uric acid levels have been linked with dyslipidemia and insulin resistance (55, 56). Choline is involved in lipoprotein synthesis and can reduce inflammatory markers (57, 58). These metabolites likely reflect compensatory metabolic responses in HC and may act as HC-specific metabolic indicators.

Pathway enrichment analysis revealed that both FH and HC shared disruptions in sphingolipid metabolism and signaling. FH patients showed upregulation of sphinganine, while HC patients upregulated both sphinganine and phytosphingosine. These sphingolipids are involved in ceramide synthesis, which regulates cholesterol storage, efflux, and inflammation (59–61). In particular, sphinganine may contribute to disease progression and serve as a potential biomarker for cardiovascular risk in both HC and FH (12, 61–65). The broader sphingolipid remodeling in HC likely reflects the added effects of insulin resistance and inflammation. Additionally, lysine degradation was enriched by increased D-alpha-hydroxyglutaric acid in FH and HC, and decreased glutaric acid in HC only. Increased D-alpha-hydroxyglutaric acid, likely reflecting mitochondrial stress and disrupted energy metabolism (66). In HC only, glutaric acid was decreased, suggesting altered metabolic flux or increased downstream utilization toward lipid biosynthesis. These shifts may influence acetyl-CoA availability, a key precursor in cholesterol and fatty acid synthesis (67).

Vitamin B6 metabolism was also altered, with pyridoxamine upregulated in both FH and HC, likely reflecting oxidative stress and inflammation (68, 69). Interestingly, 4-pyridoxic acid was only elevated in HC, suggesting increased B6 turnover driven by metabolic stress, inflammation, and insulin resistance (70, 71).

Sphinganine, D-*α*-hydroxyglutaric acid, and pyridoxamine were altered in both FH and HC, suggesting shared disruptions in lipid metabolism and oxidative stress. In contrast, dysregulation of 4-pyridoxic acid, glutaric acid, and phytosphingosine was more pronounced in HC, likely driven by metabolic and lifestyle factors such as diet, obesity, and insulin resistance.

Our data have certain limitations that should be considered when interpreting the results. The relatively small sample size may limit the statistical power and the ability to capture the full spectrum of metabolic variation among individuals with FH and HC. In addition, several detected metabolic features remained unannotated, reflecting the current limitations of metabolite databases and highlighting opportunities for the discovery of novel biomarkers. While this study focused on untargeted profiling, the functional roles of the identified metabolites were not explored, which may serve as a valuable direction for future investigations to understand the mechanistic basis of the disease.

## 5 Conclusion

This first untargeted metabolomics study in Saudi patients comparing FH and HC revealed distinct metabolic profiles. FH showed altered bile acid and steroid hormone metabolism, marked by reduced cholic acid, elevated sphinganine, and 17*α*-OHP. In contrast, HC exhibited lifestyle-related changes, including reduced UFAs, increased L-acetylcarnitine, uric acid, and choline, reflecting insulin resistance. Shared elevation of D-α-hydroxyglutaric acid and pyridoxamine suggests common mitochondrial stress, while glutaric acid, phytosphingosine, and 4-pyridoxic acid were more disrupted in HC. These metabolites may serve as biomarkers to distinguish FH from HC and support early diagnosis and personalized therapeutic strategies in different hypercholesterolemia forms.

## Data Availability

The datasets for this article are not publicly available due to concerns regarding participant/patient anonymity. Requests to access the datasets should be directed to the corresponding author.

## Footnotes

^1^ https://mycompounddiscoverer.com

## Glossary

FH: Familial Hypercholesterolemia
HC: Non-genetic Hypercholesterolemia
LDL-C: Low-Density Lipoprotein Cholesterol
LDLR: Low-Density Lipoprotein Receptor
PCSK9: Proprotein Convertase Subtilisin/Kexin Type 9
LDLRAP1: Low-Density Lipoprotein Receptor Adaptor Protein 1
17-OHP: 17α-Hydroxyprogesterone
CVDs: Cardiovascular Diseases
GPC: Glycerophosphocholine
EP: Eppendorf
BGI: Beijing Genomics Institute
AGC: Automatic Gain Control
ESI: Electrospray Ionization
BMDB: BGI Metabolome Database
PQN: Probabilistic Quotient Normalization
QC-RLSC: Quality Control-based Robust LOESS Signal Correction
CV: Coefficient of Variation
KEGG: Kyoto Encyclopedia of Genes and Genomes
HMDB: Human Metabolome Database
DEM: Differentially Expressed Metabolite

## References

[1] Lee Y Siddiqui WJ. Cholesterol levels: StatPearls. Treasure Island (FL): StatPearls Publishing (2025).

[2] Baruah DK. Familial hypercholesterolemia. Heart Views. (2021) 22:304–5. doi: 10.4103/HEARTVIEWS.HEARTVIEWS_38_21, PMID: 35330649 PMC8939378

[3] Marziliano N Medoro A Folzani S Intrieri M Reverberi C. Molecular genetics for familial hypercholesterolemia. Rev Cardiovasc Med. (2022) 23:4. doi: 10.31083/j.rcm2301004 35092196

[4] Warsy AS Al-Jaser MH Albdass A Al-Daihan S Alanazi M. Is consanguinity prevalence decreasing in Saudis?: a study in two generations. Afr Health Sci. (2014) 14:314–21. doi: 10.4314/ahs.v14i2.5, PMID: 25320579 PMC4196414

[5] Alhabib KF Al-Rasadi K Almigbal TH Batais MA Al-Zakwani I Al-Allaf FA . Familial hypercholesterolemia in the Arabian gulf region: clinical results of the Gulf FH registry. PLoS One. (2021) 16:e0251560. doi: 10.1371/journal.pone.0251560, PMID: 34086694 PMC8177652

[6] Fareed M Afzal M. Genetics of consanguinity and inbreeding in health and disease. Ann Hum Biol. (2017) 44:99–107. doi: 10.1080/03014460.2016.1265148, PMID: 27892699

[7] Al-Rasadi K Alhabib KF Al-Allaf F Al-Waili K Al-Zakwani I AlSarraf A . The Gulf familial hypercholesterolemia registry (gulf FH): design, rationale and preliminary results. Curr Vasc Pharmacol. (2020) 18:57–64. doi: 10.2174/1570161116666181005125459, PMID: 30289081 PMC7040511

[8] Watts GF Gidding S Wierzbicki AS Toth PP Alonso R Brown WV . Integrated guidance on the care of familial hypercholesterolaemia from the international FH foundation. Int J Cardiol. (2014) 171:309–25. doi: 10.1016/j.ijcard.2013.11.025, PMID: 24418289

[9] Al-Rasadi K Al-Waili K Al-Sabti HA Al-Hinai A Al-Hashmi K Al-Zakwani I . Criteria for diagnosis of familial hypercholesterolemia: a comprehensive analysis of the different guidelines, appraising their suitability in the Omani Arab population. Oman Med J. (2014) 29:85–91. doi: 10.5001/omj.2014.22, PMID: 24715932 PMC3976735

[10] Tomita M Kami K. Systems biology, metabolomics, and cancer metabolism. Science. (2012) 336:990–1. doi: 10.1126/science.1223066, PMID: 22628644

[11] Qiu S Cai Y Yao H Lin C Xie Y Tang S . Small molecule metabolites: discovery of biomarkers and therapeutic targets. Sig Transduct Target Ther. (2023) 8:132–7. doi: 10.1038/s41392-023-01399-3, PMID: 36941259 PMC10026263

[12] Du Z Li F Jiang L Li L Du Y Yu H . Metabolic systems approaches update molecular insights of clinical phenotypes and cardiovascular risk in patients with homozygous familial hypercholesterolemia. BMC Med. (2023) 21:275. doi: 10.1186/s12916-023-02967-8, PMID: 37501168 PMC10375787

[13] Zhang A Sun H Yan G Wang P Wang X. Mass spectrometry-based metabolomics: applications to biomarker and metabolic pathway research. Biomed Chromatogr. (2016) 30:7–12. doi: 10.1002/bmc.3453, PMID: 25739660

[14] Vo D-K Trinh KTL. Emerging biomarkers in metabolomics: advancements in precision health and disease diagnosis. Int J Mol Sci. (2024) 25:13190. doi: 10.3390/ijms252313190, PMID: 39684900 PMC11642057

[15] Sun W Zhang D Wang Z Sun J Xu B Chen Y . Insulin resistance is associated with Total bile acid level in type 2 diabetic and nondiabetic population: a cross-sectional study. Medicine. (2016) 95:e2778. doi: 10.1097/MD.0000000000002778, PMID: 26962776 PMC4998857

[16] Cerqueira NMFSA Oliveira EF Gesto DS Santos-Martins D Moreira C Moorthy HN . Cholesterol biosynthesis: a mechanistic overview. Biochemistry. (2016) 55:5483–506. doi: 10.1021/acs.biochem.6b00342, PMID: 27604037

[17] Alwahsh M Alejel R Hasan A Abuzaid H Al-Qirim T. The application of metabolomics in hyperlipidemia: insights into biomarker discovery and treatment efficacy assessment. Meta. (2024) 14:438. doi: 10.3390/metabo14080438, PMID: 39195534 PMC11356594

[18] Du Z Du Y Li L Sun H Hu C Jiang L . Metabolomic approach to screening homozygotes in Chinese patients with severe familial hypercholesterolemia. J Clin Med. (2023) 12:483. doi: 10.3390/jcm12020483, PMID: 36675412 PMC9861332

[19] Al Mahdi HB Shaik NA Banaganapalli B Edris S Zahed R ElSokary HA . Pathogenic LDLR variants (c.103 C>T and c.2416dup) in ligand-binding and cytosolic domains in Saudi familial hypercholesterolemia: molecular characterization and computational insights. *Computational and structural*. Biotechnol J. (2025) 27:3770–84. doi: 10.1016/j.csbj.2025.08.029, PMID: 40977904 PMC12447921

[20] Wen B Mei Z Zeng C Liu S. metaX: a flexible and comprehensive software for processing metabolomics data. BMC Bioinformatics. (2017) 18:183. doi: 10.1186/s12859-017-1579-y, PMID: 28327092 PMC5361702

[21] Di Guida R Engel J Allwood JW Weber RJM Jones MR Sommer U . Non-targeted UHPLC-MS metabolomic data processing methods: a comparative investigation of normalisation, missing value imputation, transformation and scaling. Metabolomics. (2016) 12:93. doi: 10.1007/s11306-016-1030-9, PMID: 27123000 PMC4831991

[22] Dunn WB Broadhurst D Begley P Zelena E Francis-McIntyre S Anderson N . Procedures for large-scale metabolic profiling of serum and plasma using gas chromatography and liquid chromatography coupled to mass spectrometry. Nat Protoc. (2011) 6:1060–83. doi: 10.1038/nprot.2011.335, PMID: 21720319

[23] Miname MH Santos RD. Reducing cardiovascular risk in patients with familial hypercholesterolemia: risk prediction and lipid management. Prog Cardiovasc Dis. (2019) 62:414–22. doi: 10.1016/j.pcad.2019.10.003, PMID: 31669498

[24] Ganjali S Keshavarz R Hosseini S Mansouri A Mannarino MR Pirro M . Evaluation of oxidative stress status in familial hypercholesterolemia. J Clin Med. (2021) 10:5867. doi: 10.3390/jcm10245867, PMID: 34945165 PMC8707741

[25] McGarrah RW Crown SB Zhang G-F Shah SH Newgard CB. Cardiovascular metabolomics. Circ Res. (2018) 122:1238–58. doi: 10.1161/CIRCRESAHA.117.311002, PMID: 29700070 PMC6029726

[26] Anesi A Di Minno A Calcaterra I Cavalca V Tripaldella M Porro B . An untargeted Lipidomic analysis reveals depletion of several phospholipid classes in patients with familial hypercholesterolemia on treatment with Evolocumab. Biomedicine. (2021) 9:1941. doi: 10.3390/biomedicines9121941, PMID: 34944757 PMC8698529

[27] Bochem AE Holleboom AG Romijn JA Hoekstra M Dallinga-Thie GM Motazacker MM . High density lipoprotein as a source of cholesterol for adrenal steroidogenesis: a study in individuals with low plasma HDL-C. J Lipid Res. (2013) 54:1698–704. doi: 10.1194/jlr.P033449, PMID: 23511897 PMC3646470

[28] Turcu AF Auchus RJ. Adrenal steroidogenesis and congenital adrenal hyperplasia. Endocrinol Metab Clin N Am. (2015) 44:275–96. doi: 10.1016/j.ecl.2015.02.002, PMID: 26038201 PMC4506691

[29] Lu Y Wang E Chen Y Zhou B Zhao J Xiang L . Obesity-induced excess of 17-hydroxyprogesterone promotes hyperglycemia through activation of glucocorticoid receptor. J Clin Invest. (2020) 130:3791–804. doi: 10.1172/JCI134485, PMID: 32510471 PMC7324200

[30] Honour WJ. Steroids in the laboratory and clinical practice. Netherlands: Elsevier (2023).

[31] Miller WL Bose HS. Early steps in steroidogenesis: intracellular cholesterol trafficking. J Lipid Res. (2011) 52:2111–35. doi: 10.1194/jlr.R016675, PMID: 21976778 PMC3283258

[32] Pallan PS Wang C Lei L Yoshimoto FK Auchus RJ Waterman MR . Human cytochrome P450 21A2, THE major steroid 21-hydroxylase: STRUCTURE OF THE ENZYME·PROGESTERONE SUBSTRATE COMPLEX AND RATE-LIMITING C-H BOND CLEAVAGE. J Biol Chem. (2015) 290:13128–43. doi: 10.1074/jbc.M115.646307, PMID: 25855791 PMC4505568

[33] Laue L Hoeg JM Barnes K Loriaux DL Chrousos GP. The effect of mevinolin on steroidogenesis in patients with defects in the low density lipoprotein receptor pathway. J Clin Endocrinol Metab. (1987) 64:531–5. doi: 10.1210/jcem-64-3-531, PMID: 3029155

[34] Bacila I-A Lawrence NR Badrinath SG Balagamage C Krone NP. Biomarkers in congenital adrenal hyperplasia. Clin Endocrinol. (2024) 101:300–10. doi: 10.1111/cen.14960, PMID: 37608608

[35] Therrell BL. Newborn screening for congenital adrenal hyperplasia. Endocrinol Metab Clin N Am. (2001) 30:15–30. doi: 10.1016/s0889-8529(08)70017-3, PMID: 11344933

[36] Held PK Bird IM Heather NL. Newborn screening for congenital adrenal hyperplasia: review of factors affecting screening accuracy. Int J Neonatal Screen. (2020) 6:67. doi: 10.3390/ijns6030067, PMID: 33117906 PMC7569755

[37] Subbarayan A Dattani MT Peters CJ Hindmarsh PC. Cardiovascular risk factors in children and adolescents with congenital adrenal hyperplasia due to 21-hydroxylase deficiency. Clin Endocrinol. (2014) 80:471–7. doi: 10.1111/cen.12265, PMID: 23751160 PMC4204515

[38] Tang W Norlin M Wikvall K. Glucocorticoid receptor-mediated upregulation of human CYP27A1, a potential anti-atherogenic enzyme. Biochim Biophys Acta. (2008) 1781:718–23. doi: 10.1016/j.bbalip.2008.08.005, PMID: 18817892

[39] Hofmann AF. Bile acids: the good, the bad, and the ugly. News Physiol Sci. (1999) 14:24–9. doi: 10.1152/physiologyonline.1999.14.1.24, PMID: 11390813

[40] Chiang JYL. Bile acid metabolism and signaling. Compr Physiol. (2013) 3:1191–212. doi: 10.1002/cphy.c120023, PMID: 23897684 PMC4422175

[41] Di Ciaula A Garruti G Lunardi Baccetto R Molina-Molina E Bonfrate L Wang DQ-H . Bile acid physiology. Ann Hepatol. (2017) 16:s4–s14. doi: 10.5604/01.3001.0010.5493 29080336

[42] Chiang JYL Ferrell JM. Up to date on cholesterol 7 alpha-hydroxylase (CYP7A1) in bile acid synthesis. Liver Res. (2020) 4:47–63. doi: 10.1016/j.livres.2020.05.001, PMID: 34290896 PMC8291349

[43] Li T Chiang JYL. Regulation of bile acid and cholesterol metabolism by PPARs. PPAR Res. (2009) 2009:501739. doi: 10.1155/2009/501739, PMID: 19636418 PMC2712638

[44] Shepherd J. Mechanism of action of bile acid sequestrants and other lipid-lowering drugs. Cardiology. (1989) 76:65–71. doi: 10.1159/000174548 2713876

[45] Insull W. Clinical utility of bile acid sequestrants in the treatment of dyslipidemia: a scientific review. South Med J. (2006) 99:257–73. doi: 10.1097/01.smj.0000208120.73327.db, PMID: 16553100

[46] Islam MS Sharif A Kwan N Tam KC. Bile acid Sequestrants for hypercholesterolemia treatment using sustainable biopolymers: recent advances and future perspectives. Mol Pharm. (2022) 19:1248–72. doi: 10.1021/acs.molpharmaceut.2c00007, PMID: 35333534

[47] Galgani JE Moro C Ravussin E. Metabolic flexibility and insulin resistance. Am J Physiol Endocrinol Metab. (2008) 295:E1009–17. doi: 10.1152/ajpendo.90558.2008, PMID: 18765680 PMC2584808

[48] Jump DB. Fatty acid regulation of hepatic lipid metabolism. Curr Opin Clin Nutr Metab Care. (2011) 14:115–20. doi: 10.1097/MCO.0b013e328342991c, PMID: 21178610 PMC3356999

[49] Fernandez ML West KL. Mechanisms by which dietary fatty acids modulate plasma lipids. J Nutr. (2005) 135:2075–8. doi: 10.1093/jn/135.9.2075, PMID: 16140878

[50] Borges M-C Haycock P Zheng J Hemani G Howe LJ Schmidt AF . The impact of fatty acids biosynthesis on the risk of cardiovascular diseases in Europeans and east Asians: a Mendelian randomization study. Hum Mol Genet. (2022) 31:4034–54. doi: 10.1093/hmg/ddac153, PMID: 35796550 PMC9703943

[51] Castrogiovanni P Di Rosa M Ravalli S Castorina A Guglielmino C Imbesi R . Moderate physical activity as a prevention method for knee osteoarthritis and the role of Synoviocytes as biological key. Int J Mol Sci. (2019) 20:511. doi: 10.3390/ijms20030511, PMID: 30691048 PMC6387266

[52] Ntambi JM. Regulation of stearoyl-CoA desaturase by polyunsaturated fatty acids and cholesterol. J Lipid Res. (1999) 40:1549–58. doi: 10.1016/S0022-2275(20)33401-5, PMID: 10484602

[53] Dobrzyn P Dobrzyn A Miyazaki M Cohen P Asilmaz E Hardie DG . Stearoyl-CoA desaturase 1 deficiency increases fatty acid oxidation by activating AMP-activated protein kinase in liver. Proc Natl Acad Sci USA. (2004) 101:6409–14. doi: 10.1073/pnas.0401627101, PMID: 15096593 PMC404058

[54] Bene J Hadzsiev K Melegh B. Role of carnitine and its derivatives in the development and management of type 2 diabetes. Nutr Diabetes. (2018) 8:8. doi: 10.1038/s41387-018-0017-1, PMID: 29549241 PMC5856836

[55] Liang J Jiang Y Huang Y Song W Li X Huang Y . The comparison of dyslipidemia and serum uric acid in patients with gout and asymptomatic hyperuricemia: a cross-sectional study. Lipids Health Dis. (2020) 19:31. doi: 10.1186/s12944-020-1197-y, PMID: 32127000 PMC7053114

[56] Peng T-C Wang C-C Kao T-W Chan JY-H Yang Y-H Chang Y-W . Relationship between hyperuricemia and lipid profiles in US adults. Biomed Res Int. (2015) 2015:127596. doi: 10.1155/2015/127596 25629033 PMC4299312

[57] Abbasi MSP Tousi AZ Yazdani Y Vahdat S Gharebakhshi F Nikrad N . Dietary choline and betaine intake, cardio-metabolic risk factors and prevalence of metabolic syndrome among overweight and obese adults. BMC Endocr Disord. (2023) 23:67. doi: 10.1186/s12902-023-01323-4, PMID: 36973700 PMC10041695

[58] DiBella M Thomas MS Alyousef H Millar C Blesso C Malysheva O . Choline intake as supplement or as a component of eggs increases plasma choline and reduces Interleukin-6 without modifying plasma cholesterol in participants with metabolic syndrome. Nutrients. (2020) 12:3120. doi: 10.3390/nu12103120, PMID: 33066009 PMC7600433

[59] Quinville BM Deschenes NM Ryckman AE Walia JS. A comprehensive review: sphingolipid metabolism and implications of disruption in sphingolipid homeostasis. Int J Mol Sci. (2021) 22:5793. doi: 10.3390/ijms22115793, PMID: 34071409 PMC8198874

[60] Fantini J Yahi N. Lipid metabolism and oxidation in neurons and glial cells In: Fantini J Yahi N, editors. Brain lipids in synaptic function and neurological disease. San Diego: Academic Press (2015). 53–85.

[61] Jiang X-C Li Z. Sphingolipids and cholesterol. Adv Exp Med Biol. (2022) 1372:1–14. doi: 10.1007/978-981-19-0394-6_1, PMID: 35503170 PMC9251731

[62] Berkowitz L Salazar C Ryff CD Coe CL Rigotti A. Serum sphingolipid profiling as a novel biomarker for metabolic syndrome characterization. Front Cardiovasc Med. (2022) 9:1092331. doi: 10.3389/fcvm.2022.1092331, PMID: 36578837 PMC9791223

[63] Ramos-Molina B Rossell J Pérez-Montes de Oca A Pardina E Genua I Rojo-López MI . Therapeutic implications for sphingolipid metabolism in metabolic dysfunction-associated steatohepatitis. Front Endocrinol. (2024) 15:1400961. doi: 10.3389/fendo.2024.1400961, PMID: 38962680 PMC11220194

[64] Borodzicz-Jażdżyk S Jażdżyk P Łysik W Cudnoch-Jȩdrzejewska A Czarzasta K. Sphingolipid metabolism and signaling in cardiovascular diseases. Front Cardiovasc Med. (2022) 9:915961. doi: 10.3389/fcvm.2022.915961, PMID: 36119733 PMC9471951

[65] Juhász L Lőrincz H Szentpéteri A Nádró B Varga É Paragh G . Sphingosine 1-phosphate and apolipoprotein M levels and their correlations with inflammatory biomarkers in patients with untreated familial hypercholesterolemia. Int J Mol Sci. (2022) 23:14065. doi: 10.3390/ijms232214065, PMID: 36430543 PMC9697457

[66] Wang Z Liu H. Roles of lysine methylation in glucose and lipid metabolism: functions, regulatory mechanisms, and therapeutic implications. Biomolecules. (2024) 14:862. doi: 10.3390/biom14070862, PMID: 39062577 PMC11274642

[67] Martínez-Reyes I Chandel NS. Mitochondrial TCA cycle metabolites control physiology and disease. Nat Commun. (2020) 11:102. doi: 10.1038/s41467-019-13668-3, PMID: 31900386 PMC6941980

[68] Vekic J Stromsnes K Mazzalai S Zeljkovic A Rizzo M Gambini J. Oxidative stress, Atherogenic dyslipidemia, and cardiovascular risk. Biomedicine. (2023) 11:2897. doi: 10.3390/biomedicines11112897, PMID: 38001900 PMC10669174

[69] Ramis R Ortega-Castro J Caballero C Casasnovas R Cerrillo A Vilanova B . How does Pyridoxamine inhibit the formation of advanced glycation end products? The role of its primary antioxidant activity. Antioxidants. (2019) 8:344. doi: 10.3390/antiox8090344, PMID: 31480509 PMC6770850

[70] Obeid R Geisel J Nix WA. 4-Pyridoxic acid/pyridoxine ratio in patients with type 2 diabetes is related to global cardiovascular risk scores. Diagnostics. (2019) 9:28. doi: 10.3390/diagnostics9010028, PMID: 30845778 PMC6468858

[71] Mascolo E Vernì F. Vitamin B6 and diabetes: relationship and molecular mechanisms. Int J Mol Sci. (2020) 21:3669. doi: 10.3390/ijms21103669, PMID: 32456137 PMC7279184

