# Correction: Untargeted metabolomics reveals distinct biomarkers and metabolic alterations in familial and non-genetic hypercholesterolemia in Saudi patients

**DOI:** 10.3389/fmed.2026.1817633

**Published:** 2026-05-11

**Authors:** Hadiah Bassam Al Mahdi, Noor Ahmad Shaik, Zuhier Awan, Hussam Daghistani, Faisal Alandejani, Kawthar Saad Alghamdi, Ahmad A. Obaid, Rawabi Zahed, Reem Nabil Hassan, Sherif Edris, Babajan Banaganapalli, Abdulrahman Mujalli

**Affiliations:** 1 Department of Biological Sciences, Faculty of Science, King Abdulaziz University, Jeddah, Saudi Arabia; 2 Department of Genetic Medicine, Faculty of Medicine, King Abdulaziz University, Jeddah, Saudi Arabia; 3 Princess Al-Jawhara Al-Brahim Centre of Excellence in Research of Hereditary Disorders, King Abdulaziz University, Jeddah, Saudi Arabia; 4 Department of Clinical Biochemistry, Faculty of Medicine, King Abdulaziz University, Jeddah, Saudi Arabia; 5 Regenerative Medicine Unit, King Fahd Medical Research Center, King Abdulaziz University, Jeddah, Saudi Arabia; 6 Department of Biology, College of Science, University of Hafr Al Batin, Hafar Al Batin, Saudi Arabia; 7 Department of Physiology, Faculty of Medicine, Umm Al-Qura University, Makkah, Saudi Arabia; 8 Department of Clinical Laboratory Sciences, Faculty of Applied Medical Sciences, Umm Al-Qura University, Makkah, Saudi Arabia

**Keywords:** familial hypercholesterolemia, untargeted metabolomics, KEGG database, biomarkers, cholic acid

Author “Noor Ahmad Shaik” was erroneously omitted as an equal contributing first author. The equal contribution phrase “These authors share first authorship” has been added to authors “Hadiah Bassam Al Mahdi” and “Noor Ahmad Shaik”.

The funder ¨King Abdulaziz University¨, ¨No. KAUKUJRP-1M-2021¨ was erroneously omitted. The correct **Funding** statement reads:

¨The author(s) declared that financial support was received for this work and/or its publication. This publication is based upon work supported by the Khalifa University (KU) and King Abdulaziz University (KAU) Join Research Program Award No. KAUKUJRP-1M-2021. ¨

Affiliation “Department of Research and Development, Al Borg Diagnostics, Jeddah, Saudi Arabia” was erroneously included in the affiliation list. This affiliation has now been removed from the article.

The original version of this article has been updated.

