# Supplementary material for: Untargeted metabolomics reveals distinct biomarkers and metabolic alterations in familial and non-genetic hypercholesterolemia in Saudi patients
Source: Front Med (Lausanne). 2025 Sep 26;12:1670282. doi: 10.3389/fmed.2025.1670282 (PMC12511148; doi:10.3389/fmed.2025.1670282)
Supplement: Supplementary file 1 [file Data_Sheet_1.docx]

Supplementary Figure 1: Base peak chromatograms (BPC) for metabolite analysis in positive and negative ion modes. **(A)** Positive ion mode and **(B)** Negative ion mode chromatograms illustrating the separation and detection of metabolites across a retention time of 0.00–15.00 minutes. The X-axis represents retention time (min), while the Y-axis indicates relative ion abundance. In the positive ion mode, prominent peaks at 7.36, 9.71, and 12.75 mins correspond to high-intensity metabolites, with earlier peaks (at 0.88 and 2.67 mins) representing polar compounds and later peaks reflecting hydrophobic metabolites. Similarly, the negative ion mode provides complementary metabolic insights, capturing acidic and polar metabolites with significant peaks at 9.73, 10.32, and 12.83 mins. Analysis was performed using the HM400 method for comprehensive metabolite profiling.

Supplementary Figure 2: Metabolite classification and KEGG pathway analysis. **(A)** The bar chart illustrates metabolite classifications, where the X-axis indicates the number of metabolites, and the Y-axis lists the classification categories. **(B)** The ring chart depicts KEGG pathway annotations, where the segments represent the number of annotated metabolites within specific KEGG pathways.

**Supplementary Table 1:** Sample information across study groups.

| **Sample ID** | **LDL-C (mg/dL)** | **Gender** | **Age** |
| --- | --- | --- | --- |
| **Familial Hypercholesterolemia (FH)** | | | |
| FH1 | 369 | M | 25 |
| FH2 | 386 | F | 26 |
| FH3 | 295 | F | 18 |
| FH4 | 296 | F | 20 |
| FH5 | 579 | M | 28 |
| FH6 | 286 | F | 22 |
| FH7 | 360 | F | 43 |
| **Hypercholesterolemia (HC)** | | | |
| HC1 | 143 | M | 28 |
| HC2 | 134 | M | 23 |
| HC3 | 137 | F | 31 |
| HC4 | 143 | F | 24 |
| **Healthy** | | | |
| C1 | 90 | F | 22 |
| C2 | 85 | F | 19 |
| C3 | 86 | F | 16 |
| C4 | 100 | F | 37 |
| C5 | 112 | F | 16 |

*C = Healthy

**Supplementary Table 2:** The significant DEMs included in the enrichment analysis

| **FH vs. Healthy** | | | | | |
| --- | --- | --- | --- | --- | --- |
| Name | KEGG ID | HMDB ID | Log2FC | P-value | FC |
| 5alpha-Pregnan-3beta,20beta-diol 3-sulfate |  | HMDB0240579 | -3.06462 | 0.02210108 | 0.119524 |
| 2(R)-hydroxydocosanoic acid |  | HMDB0061660 | -2.57164 | 0.03971311 | 0.168213 |
| Testosterone decanoate | C14607 | HMDB0002998 | -2.28396 | 0.03149451 | 0.205333 |
| PC(18:0/20:3(5Z,8Z,11Z)) |  | HMDB0008046 | -1.48421 | 0.0032139 | 0.357443 |
| PC(14:0/18:2(9Z,12Z)) |  | HMDB0007874 | -1.38571 | 0.02546475 | 0.382702 |
| PC(16:0/16:1(9Z)) |  | HMDB0007969 | -1.30588 | 0.00921196 | 0.404475 |
| PC(16:0/18:3(9Z,12Z,15Z)) |  | HMDB0007975 | -1.25259 | 0.00799926 | 0.419693 |
| PC(16:0/20:3(8Z,11Z,14Z)) | C00157 | HMDB0007981 | -1.0207 | 0.03430074 | 0.492878 |
| PC(18:0/18:2(9Z,12Z)) |  | HMDB0008039 | -0.8436 | 0.03986951 | 0.55725 |
| PC(16:0/18:1(9Z)) |  | HMDB0007972 | -0.78813 | 0.04961981 | 0.579094 |
| Indoleacetic acid | C00954 | HMDB0000197 | -0.68973 | 0.03267833 | 0.619971 |
| Isoquinoline | C06323 | HMDB0034244 | -0.61782 | 0.04342374 | 0.651655 |
| Asymmetric dimethylarginine | C03626 | HMDB0001539 | 0.293919 | 0.04600092 | 1.225966 |
| 5'-Methylthioadenosine | C00170 | HMDB0001173 | 0.390349 | 0.00692864 | 1.31071 |
| Uric acid | C00366 | HMDB0000289 | 0.407802 | 0.01715921 | 1.326663 |
| Sphinganine | C00836 | HMDB0000269 | 0.414687 | 0.04437729 | 1.33301 |
| Ribitol | C00474 | HMDB0000508 | 0.423582 | 0.00165193 | 1.341253 |
| N-Acetylalanine |  | HMDB0000766 | 0.460011 | 0.02149316 | 1.375552 |
| (R)-3-Hydroxy myristic acid | C22001 | HMDB0010731 | 0.524152 | 0.01058683 | 1.438088 |
| Pyridoxamine | C00534 | HMDB0001431 | 0.569926 | 0.01174634 | 1.484448 |
| 1-Methylhistamine | C05127 | HMDB0000898 | 0.595102 | 0.00900294 | 1.510579 |
| Hydroxyphenyllactic acid | C03672 | HMDB0000755 | 0.608933 | 0.00050609 | 1.525131 |
| 17alpha-Hydroxyprogesterone | C01176 | HMDB0000374 | 0.622189 | 0.01838874 | 1.539209 |
| L-Proline | C00148 | HMDB0000162 | 0.653248 | 0.01767099 | 1.572705 |
| Cortolone-3-glucuronide |  | HMDB0010320 | 0.804882 | 0.00697397 | 1.747003 |
| LysoPC(P-16:0/0:0) |  | HMDB0010407 | 0.916402 | 0.0017624 | 1.887403 |
| D-alpha-Hydroxyglutaric acid | C01087 | HMDB0000606 | 1.058644 | 0.03079193 | 2.082973 |
| Valylphenylalanine |  | HMDB0029134 | 1.413746 | 0.04943715 | 2.66428 |
| delta-Valerolactam |  | HMDB0011749 | 1.471552 | 0.04402608 | 2.7732 |
| 3-Carboxy-4-methyl-5-propyl-2-furanpropionic acid |  | HMDB0061112 | 2.057614 | 0.02072311 | 4.162973 |
| 3-(3,4,5-Trimethoxyphenyl)propanoic acid |  | HMDB0030254 | 2.057614 | 0.02072311 | 4.162973 |
| Trimethylamine N-oxide | C01104 | HMDB0000925 | 2.608677 | 0.0242297 | 6.099439 |
| **HC vs. Healthy** | | | | | |
| Name | KEGG ID | HMDB ID | Log2FC | P-value | FC |
| 2,4-Dihydroxyacetophenone 5-sulfate |  | HMDB0041646 | -5.90013 | 0.02868101 | 0.016745 |
| Glutaric acid | C00489 | HMDB0000661 | -3.4353 | 0.01175616 | 0.092442 |
| Traumatic acid | C16308 | HMDB0000933 | -2.81414 | 0.01250447 | 0.142187 |
| SM(d18:1/16:0) |  | HMDB0010169 | -2.69836 | 0.04521217 | 0.154068 |
| 3-Hydroxy-3-methylbutanoic acid | C20827 | HMDB0000754 | -2.69106 | 0.01672997 | 0.15485 |
| PC(P-18:0/22:6(4Z,7Z,10Z,13Z,16Z,19Z)) |  | HMDB0011262 | -2.44136 | 0.00300815 | 0.18411 |
| SM(d18:1/18:1(11Z)) |  | HMDB0012100 | -2.37984 | 0.02625698 | 0.192131 |
| PC(O-18:0/20:4(8Z,11Z,14Z,17Z)) |  | HMDB0013420 | -2.34781 | 0.01996065 | 0.196444 |
| PC(P-16:0/18:2(9Z,12Z)) |  | HMDB0011211 | -2.24534 | 0.04485979 | 0.210904 |
| PC(16:0/20:3(8Z,11Z,14Z)) | C00157 | HMDB0007981 | -2.15446 | 0.0389892 | 0.224617 |
| PC(18:1(9Z)/22:6(4Z,7Z,10Z,13Z,16Z,19Z)) |  | HMDB0008123 | -2.14656 | 0.02355874 | 0.22585 |
| PC(16:0/22:6(4Z,7Z,10Z,13Z,16Z,19Z)) |  | HMDB0007991 | -2.10849 | 0.04507083 | 0.23189 |
| PC(18:2(9Z,12Z)/18:2(9Z,12Z)) |  | HMDB0008138 | -1.88981 | 0.03968331 | 0.269843 |
| PC(16:0/18:2(9Z,12Z)) |  | HMDB0007973 | -1.84913 | 0.0444925 | 0.27756 |
| Adipic acid | C06104 | HMDB0000448 | -1.84393 | 0.00115185 | 0.278561 |
| PC(16:0/18:3(9Z,12Z,15Z)) |  | HMDB0007975 | -1.78994 | 0.04186357 | 0.289184 |
| 3-Hydroxybutyric acid | C01089 | HMDB0000011 | -1.76179 | 0.02081055 | 0.294882 |
| SM(d18:1/14:0) |  | HMDB0012097 | -1.64869 | 0.01871449 | 0.318929 |
| Octadecanamine |  | HMDB0029586 | -1.60234 | 0.03030187 | 0.329343 |
| Methylglutaric acid |  | HMDB0000752 | -1.57904 | 0.04364173 | 0.334703 |
| Myristoleoylcarnitine |  | HMDB0240588 | -1.23523 | 0.01466679 | 0.424775 |
| cis-5-Tetradecenoylcarnitine |  | HMDB0002014 | -1.19037 | 0.01249609 | 0.438191 |
| LysoPE(0:0/18:0) |  | HMDB0011129 | -1.16008 | 0.01280891 | 0.447488 |
| Myristoleic acid | C08322 | HMDB0002000 | -1.12926 | 0.02067271 | 0.457152 |
| 2-Arachidonoyl glycerol | C13856 | HMDB0004666 | -1.10885 | 0.01950032 | 0.463665 |
| 11(Z),14(Z)-Eicosadienoic acid | C16525 | HMDB0005060 | -1.04309 | 0.0148696 | 0.485287 |
| Oleic acid | C00712 | HMDB0000207 | -0.96888 | 0.00179512 | 0.510902 |
| LysoPC(O-18:0/0:0) | C04317 | HMDB0011149 | -0.92763 | 0.02681997 | 0.525721 |
| Elaidic acid | C01712 | HMDB0000573 | -0.92175 | 0.00235001 | 0.527869 |
| Indoleacetic acid | C00954 | HMDB0000197 | -0.86655 | 0.00701676 | 0.548455 |
| L-Acetylcarnitine | C02571 | HMDB0000201 | -0.81769 | 0.01225737 | 0.567348 |
| Isoquinoline | C06323 | HMDB0034244 | -0.80184 | 0.00774275 | 0.573615 |
| Linoleic acid | C01595 | HMDB0000673 | -0.76168 | 0.00229598 | 0.58981 |
| 4-Heptenoic acid |  | HMDB0033793 | -0.60732 | 0.04353953 | 0.656417 |
| LysoPI(18:0/0:0) |  | HMDB0240261 | -0.5015 | 0.02443467 | 0.706372 |
| Betaine |  | HMDB0000043 | 0.401027 | 0.0246868 | 1.320448 |
| Sphinganine | C00836 | HMDB0000269 | 0.434479 | 0.02291529 | 1.351423 |
| Creatinine | C00791 | HMDB0000562 | 0.441614 | 0.01697968 | 1.358123 |
| DL-Alanine | C01401 | HMDB0062251 | 0.496543 | 0.02950789 | 1.410829 |
| Uric acid | C00366 | HMDB0000289 | 0.496648 | 0.03083121 | 1.410931 |
| Citroflex 2 | D06228 | HMDB0034263 | 0.511647 | 0.01734536 | 1.425677 |
| D-alpha-Hydroxyglutaric acid | C01087 | HMDB0000606 | 0.555208 | 0.01939915 | 1.46938 |
| Pyridoxamine | C00534 | HMDB0001431 | 0.607339 | 0.04946585 | 1.523446 |
| N-Acetylalanine |  | HMDB0000766 | 0.610158 | 0.01402714 | 1.526427 |
| 5'-Methylthioadenosine | C00170 | HMDB0001173 | 0.640858 | 0.03973487 | 1.559256 |
| Saxitoxin | C13757 | HMDB0029368 | 0.679557 | 0.03053208 | 1.601648 |
| Choline | C00114 | HMDB0000097 | 0.727298 | 0.01882782 | 1.655536 |
| 4-Methylcatechol 2-sulfate |  | HMDB0240461 | 0.742225 | 0.03882588 | 1.672754 |
| L-Histidine | C00135 | HMDB0000177 | 0.790929 | 0.01153909 | 1.730189 |
| 4-Pyridoxic acid | C00847 | HMDB0000017 | 0.812074 | 0.00593071 | 1.755734 |
| Aniline | C00292 | HMDB0003012 | 0.855623 | 0.00390433 | 1.80954 |
| Cuminaldehyde | C06577 | HMDB0002214 | 0.874849 | 0.03033614 | 1.833817 |
| 5-Hydroxyindoleacetic acid | C05635 | HMDB0000763 | 0.877846 | 0.02668717 | 1.83763 |
| LysoPC(14:0/0:0) |  | HMDB0010379 | 0.889931 | 0.01216725 | 1.853088 |
| Phytosphingosine | C12144 | HMDB0004610 | 0.903811 | 0.02941136 | 1.871002 |
| L-Kynurenine | C00328 | HMDB0000684 | 0.975943 | 0.00588298 | 1.966926 |
| N1-Methyl-2-pyridone-5-carboxamide | C05842 | HMDB0004193 | 1.25743 | 0.04889417 | 2.390695 |
| L,L-Cyclo(leucylprolyl) |  | HMDB0034276 | 1.657081 | 0.00770213 | 3.153777 |
| 2,6-Dihydroxybenzoic acid | C21298 | HMDB0013676 | 1.847172 | 0.03375311 | 3.597943 |
| Trimethylamine N-oxide | C01104 | HMDB0000925 | 1.862759 | 0.00290365 | 3.637024 |
| Eucalyptol | C09844 | HMDB0004472 | 3.306473 | 0.03607825 | 9.893445 |
| **FH vs. HC** | | | | | |
| Name | KEGG ID | HMDB ID | Log2FC | P-value | FC |
| Chenodeoxycholic acid 3-sulfate |  | HMDB0002586 | -2.0042 | 0.01829798 | 0.249273 |
| Menadione | C05377 | HMDB0001892 | -1.78121 | 0.01626824 | 0.290938 |
| Corchorifatty acid F |  | HMDB0035919 | -1.34087 | 0.029353 | 0.394781 |
| LysoPE(0:0/16:1(9Z)) |  | HMDB0011474 | -1.1355 | 0.01503823 | 0.455177 |
| Quinaldic acid | C06325 | HMDB0000842 | -0.71328 | 0.04591811 | 0.609932 |
| Saxitoxin | C13757 | HMDB0029368 | -0.69896 | 0.03619845 | 0.616015 |
| Cholic acid | C00695 | HMDB0000619 | -0.43066 | 0.0076687 | 0.741922 |
| D-Serine | C00740 | HMDB0003406 | 0.511466 | 0.0123547 | 1.425498 |
| D-Aspartic acid | C00402 | HMDB0006483 | 0.549816 | 0.03922564 | 1.463899 |
| LysoPC(P-16:0/0:0) |  | HMDB0010407 | 0.782466 | 0.00682967 | 1.720069 |
| Fluoxetine |  | HMDB0014615 | 0.862103 | 0.04113709 | 1.817686 |
| Allopregnanolone | C13712 | HMDB0001449 | 1.027784 | 0.04589343 | 2.038891 |
| 4-Heptenoic acid |  | HMDB0033793 | 1.247108 | 0.00971354 | 2.373652 |
| LysoPC(O-18:0/0:0) | C04317 | HMDB0011149 | 1.270073 | 0.0131418 | 2.411738 |
| 16alpha-Hydroxy DHEA 3-sulfate |  | HMDB0062544 | 1.291624 | 0.03155245 | 2.448035 |
| Crotonic acid | C01771 | HMDB0010720 | 1.445615 | 0.00370349 | 2.723789 |
| LysoPE(0:0/18:0) |  | HMDB0011129 | 1.577269 | 0.02307662 | 2.984045 |
| Glutaric acid | C00489 | HMDB0000661 | 3.018746 | 0.03200927 | 8.10463 |
| alpha-Aspartylphenylalanine |  | HMDB0000706 | 3.07802 | 0.01596335 | 8.444546 |
| 2-Anisic acid |  | HMDB0032604 | 3.132822 | 0.01222856 | 8.771491 |
| Phenol | C15584 | HMDB0000228 | 3.420327 | 0.00563906 | 10.70584 |
| 4-Methylphenol | C01468 | HMDB0001858 | 4.46417 | 0.0062101 | 22.07237 |

**Supplementary Table 3:** Enrichment analysis-KEGG pathway score plot and Abundance score

| **Pathway Information** | | | | | **Bubble map** | | **Pathway Score** | |
| --- | --- | --- | --- | --- | --- | --- | --- | --- |
| **Pathway ID** | **Pathway** | **Count** | **Down** | **Up** | **DA score** | **State** | **Color  (P-value)** | **X-axis (RichFactor)** |
| **FH vs. Healthy** | | | | | | | | |
| map00970 | Aminoacyl-tRNA biosynthesis | 1 | 0 | 1 | 1 | Up | 0.026941 | 0.0416667 |
| map04927 | Cortisol synthesis and secretion | 1 | 0 | 1 | 1 | Up | 0.013558 | 0.0833333 |
| map04934 | Cushing syndrome | 1 | 0 | 1 | 1 | Up | 0.014679 | 0.0769231 |
| map04978 | Mineral absorption | 1 | 0 | 1 | 1 | Up | 0.032466 | 0.0344828 |
| map04913 | Ovarian steroidogenesis | 1 | 0 | 1 | 1 | Up | 0.025832 | 0.0434783 |
| map00740 | Riboflavin metabolism | 1 | 0 | 1 | 1 | Up | 0.026941 | 0.0416667 |
| map00600 | Sphingolipid metabolism | 1 | 0 | 1 | 1 | Up | 0.012434 | 0.0909091 |
| map04071 | Sphingolipid signaling pathway | 1 | 0 | 1 | 1 | Up | 0.01131 | 0.1 |
| map00750 | Vitamin B6 metabolism | 1 | 0 | 1 | 1 | Up | 0.032466 | 0.0344828 |
| map04977 | Vitamin digestion and absorption | 1 | 0 | 1 | 1 | Up | 0.033568 | 0.0333333 |
| **HC vs. Healthy** | | | | | | | | |
| map05143 | African trypanosomiasis | 1 | 0 | 1 | 1 | Up | 0.026016 | 0.1428571 |
| map04976 | Bile secretion | 2 | 0 | 2 | 1 | Up | 0.024892 | 0.0215054 |
| map01040 | Biosynthesis of unsaturated fatty acids | 3 | 3 | 0 | -1 | Down | 0.006238 | 0.0434783 |
| map05231 | Choline metabolism in cancer | 1 | 0 | 1 | 1 | Up | 0.026016 | 0.2 |
| map04725 | Cholinergic synapse | 1 | 0 | 1 | 1 | Up | 0.006238 | 0.1 |
| map00310 | Lysine degradation | 2 | 1 | 1 | 0 | Down | 0.006238 | 0.0444444 |
| map00600 | Sphingolipid metabolism | 2 | 0 | 2 | 1 | Up | 0.000367 | 0.1818182 |
| map04071 | Sphingolipid signaling pathway | 1 | 0 | 1 | 1 | Up | 0.006238 | 0.1 |
| map00380 | Tryptophan metabolism | 3 | 1 | 2 | 0.33 | Up | 0.001349 | 0.0361446 |
| map00750 | Vitamin B6 metabolism | 2 | 0 | 2 | 1 | Up | 0.002627 | 0.0689655 |
| **FH vs. HC** | | | | | | | | |
| map00250 | Alanine, aspartate and glutamate metabolism | 1 | 0 | 1 | 1 | Up | 0.021563 | 0.0357143 |
| map00470 | D-Amino acid metabolism | 2 | 0 | 2 | 1 | Up | 0.001129 | 0.0307692 |
| map00071 | Fatty acid degradation | 1 | 0 | 1 | 1 | Up | 0.029916 | 0.025641 |
| map00260 | Glycine, serine and threonine metabolism | 1 | 0 | 1 | 1 | Up | 0.033691 | 0.0227273 |
| map00310 | Lysine degradation | 1 | 0 | 1 | 1 | Up | 0.034445 | 0.0222222 |
| map00120 | Primary bile acid biosynthesis | 1 | 1 | 0 | -1 | Down | 0.03595 | 0.0212766 |
| map04974 | Protein digestion and absorption | 1 | 0 | 1 | 1 | Up | 0.035198 | 0.0217391 |
| map04742 | Taste transduction | 1 | 0 | 1 | 1 | Up | 0.024608 | 0.03125 |
| map04977 | Vitamin digestion and absorption | 1 | 1 | 0 | -1 | Down | 0.023087 | 0.0333333 |
